# Association between bile acid metabolism and bone mineral density in postmenopausal women

**DOI:** 10.6061/clinics/2020/e1486

**Published:** 2020-03-09

**Authors:** Yu-Xiao Zhao, Yu-Wen Song, Liang Zhang, Feng-Jie Zheng, Xue-Meng Wang, Xiang-Hua Zhuang, Fei Wu, Jian Liu

**Affiliations:** I Nanchang University Queen Mary School, Nanchang, Jiangxi, 330031, China; II Department of Endocrinology, The Second Hospital of Shandong University, Jinan, Shandong, 250033, China; III Department of Emergency Medicine, The First Affiliated Hospital of Nanchang University, Nanchang, Jiangxi, 330006, China

**Keywords:** Postmenopausal Osteoporosis, Bile Acid Metabolism, FGF19

## Abstract

**OBJECTIVES:**

Previous studies have not shown any correlation between bile acid metabolism and bone mineral density (BMD) in women with postmenopausal osteoporosis. Thus, the current study evaluated the association between bile acid levels as well as BMD and bone turnover marker levels in this group of women.

**METHODS:**

This single-center cross-sectional study included 150 postmenopausal Chinese women. According to BMD, the participants were divided into three groups: osteoporosis group, osteopenia group, and healthy control group. Serum bile acid, fibroblast growth factor 19 (FGF19), and bone turnover biomarker levels were assessed. Moreover, the concentrations of parathyroid hormone, 25-hydroxy vitamin D [25(OH)D], procollagen type I N-peptide (P1NP), and beta-CrossLaps of type I collagen containing cross-linked C-terminal telopeptide (β-CTX) were evaluated. The BMD of the lumbar spine and proximal femur were examined via dual-energy X-ray absorptiometry.

**RESULTS:**

The serum total bile acid levels in the osteoporosis and osteopenia groups (5.28±1.56 and 5.31±1.56 umol/L, respectively) were significantly lower than that in the healthy control group (6.33±2.04 umol/L; *p*=0.002 and 0.018, respectively). Serum bile acid level was positively associated with the BMD of the lumbar spine, femoral neck, and total hip. However, it negatively correlated with β-CTX concentration. Moreover, no correlation was observed between bile acid and P1NP levels, and the levels of the other biomarkers that were measured did not differ between the groups.

**CONCLUSION:**

Serum bile acid was positively correlated with BMD and negatively correlated with bone turnover biomarkers reflecting bone absorption in postmenopausal women. Thus, bile acid may play an important role in bone metabolism.

## INTRODUCTION

Osteoporosis is the most common bone disease, and it is characterized by low bone mass, destruction of bone microstructure, and increased bone fragility. An epidemiological survey conducted in 2018 has shown that the prevalence of osteoporosis in the population aged over 50 years in China is 19.2%. Moreover, the prevalence rate of osteoporosis in men was 6.0%, and that in women is as high as 32.1%. Meanwhile, the prevalence rate of osteoporosis among women aged over 65 years is as high as 51.6%, and this rate is significantly higher than that in women in European and American countries (1,2). Osteoporosis can easily lead to fracture and other complications. Thus, it is one of the main causes of disability and death among elderly individuals (3).

Bile acid, which is synthesized and secreted by liver cells, is a component of bile and an important signaling molecule that plays a role in glucose and lipid metabolism, energy metabolism, and intestinal flora regulation (4,5). Recent studies have found that bile acid has protective effects on the bones (6). Moreover, it transduces signals mainly by activating the nuclear receptor, farnesoid X receptor (FXR), membrane receptor, and G protein-coupled bile acid receptor 5 (TGR5) (7). A previous study has revealed the occurrence of rapid and severe bone loss after ovariectomy in FXR knockout mice (8). FXR gene knockout has bidirectional effects on the bones. That is, it does not only promote the differentiation and function of osteoclasts but also inhibits the differentiation and function of osteoblasts. Moreover, a research has found that bile acid may have therapeutic effects against osteoporosis. In this study, the bone mineral density (BMD) of the mouse models of osteoporosis significantly increased after the administration of a bile acid receptor agonist. However, to date, only few clinical studies have shown the association between bile acid metabolism and BMD. Moreover, the underlying mechanisms remain unknown (9,10).

Therefore, in this study, we aimed to examine the bile acid metabolism of patients with postmenopausal osteoporosis by evaluating the levels of total bile acid and fibroblast growth factor 19 (FGF19). Moreover, we analyzed the correlation between bile acid metabolism as well as bone turnover marker levels and BMD and the mechanisms underlying the effect of bile acid on bone metabolism.

## SUBJECTS AND METHODS

### Study population

The study was approved by the Scientific Research Ethics Committee of The Second Hospital of Shandong University (KYLL-2018[KJ]P-001). All participants provided a written informed consent for medical research.

This was a single-center cross-sectional study. Altogether, 150 postmenopausal women who were initially treated in the Department of Endocrinology of the Second Hospital of Shandong University from January 2019 to May 2019 were enrolled. According to BMD, the participants were divided into three groups, which were as follows: osteoporosis group, osteopenia group, and healthy control group. The inclusion criteria were (1) women who had been postmenopausal for more than 1 year and (2) age over 50 years. Meanwhile, the exclusion criteria were the presence of (1) metabolic or genetic bone diseases, such as rickets, osteomalacia, fibrous dysplasia of the bone, and hyperparathyroidism (2) endocrine diseases affecting bone metabolism, including hyperthyroidism, diabetes, and Cushing syndrome; (3) diseases affecting bone metabolism, such as chronic liver and kidney diseases, severe gastrointestinal diseases, neoplastic diseases, and autoimmune diseases; (4) use of long-term medication or hormone replacement therapy affecting bone metabolism; and (5) new-onset fractures within the last 3 months.

### Laboratory tests

A detailed medical history was obtained, and information about the patients’ age, height, and weight and other data were obtained. After the participants fasted overnight, blood samples were collected to assess the levels of biochemical indicators, including calcium (Ca), phosphorus (P), total alkaline phosphatase (TALP), alanine aminotransferase (ALT), creatinine (Cr), 25-hydroxy vitamin D [25(OH)D], intact parathyroid hormone (PTH), procollagen type I N-peptide (P1NP), β-CrossLaps of type I collagen containing cross-linked C-terminal telopeptide (β-CTX), total bile acid, and FGF19. The Ca, P, TALP, PTH, ALT, Cr, and total bile acid concentrations were measured using an automatic biochemistry analyzer in the Laboratory Medicine Center of the Second Hospital of Shandong University. The concentrations of other compounds, including 25(OH)D, P1NP, β-CTX, and FGF19 (Cusabio, Wuhan, China), were determined using an enzyme-linked immunosorbent assay based on well-established methods (10).

### Measurement of BMD

Dual energy X-ray absorptiometry was used to measure the areal BMD (unit g/cm2) of lumbar spines 1-4, femoral neck, and total hip using the Lunar Prodigy device (GE Lunar Corp.). Trained technicians from the Department of Radiology performed the tests. Before using the instruments, an accuracy test was conducted with a general phantom to ensure the stability of the system. If there were serious bone deformities or surgical implants in the measurement area, the BMD results were considered unreliable and were not included in the statistical analysis.

### Statistical methods

The general characteristics of the patients were expressed using descriptive statistics. All continuous variables were tested for normality and were expressed as mean±standard deviation (SD) or percentages unless stated otherwise. The three groups were compared using chi-square test and one-way analysis of variance. The correlation between bile acid metabolism and BMD was analyzed using Spearman correlation coefficient test.

*p*-values <0.05 were considered statistically significant. All statistical analyses were performed using the Statistical Package for the Social Sciences version 24.0 (SPSS Inc., Chicago, IL, the USA).

## RESULTS

### General characteristics of the participants

Altogether, 150 postmenopausal women aged over 50 years (mean age: 59.0±4.3 [range: 50-68] years) were included, and 52, 75, and 23 patients were classified under the osteoporosis, osteopenia, and control groups, respectively. 1 - Osteoporosis: Presence of fragility fractures in the absence of other metabolic bone disorders or a BMD T-score of -2.5 or lower in the lumbar spine, femoral neck or total hip measured by DXA; 2 - Osteopenia: -2.5<BMDT-score<-1.0 in the lumbar spine, femoral neck or total hip measured by DXA; 3 - control group: BMD T-score≥-1.0 in the lumbar spine, femoral neck or total hip measured by DXA. No significant difference was observed in terms of height, weight, body mass index, and levels of blood lipid, Ca, P, PTH, 25(OH)D, and the other indicators between the three groups (Tables 1, 2). In terms of bone turnover biomarker levels, no significant difference was observed in TALP or P1NP level between the three groups (Table 2). The serum β-CTX levels of the three groups were 572.3±154.4, 543.6±96.3, and 479.4±83.2 pg/mL, respectively, thereby indicating a gradual decrease in the values and a significant difference in the pair-wise comparisons between the three groups (all *p*<0.05). The healthy control group had the highest BMD and T values of the lumbar spine, femoral neck, and total hip, followed by the osteopenia and osteoporosis groups, and significant differences were observed in the pair-wise comparisons between the three groups (all *p*<0.05) (Table 2).

### Changes in the level of bile acid metabolism-related indicators in patients with osteoporosis

The serum bile acid levels were lower in the osteoporosis and osteopenia groups (5.28±1.56 and 5.31±1.56 umol/L, respectively) than in the control group (6.33±2.04 umol/L; *p*=0.002 and 0.018, respectively). The FGF19 levels were lower in the osteoporosis and osteopenia groups (5105.0±2567.1 and 4915.1±2413.2 pg/mL, respectively) than in the healthy control group (7107.7±4240.1 pg/mL; *p*=0.006 and 0.016, respectively).

### Correlation between bile acid metabolism as well as bone turnover indicator levels and BMD

Spearman’s correlation analysis revealed that serum bile acid level positively correlated to lumbar spine BMD (R=0.249, *p*=0.012), T value of the lumbar spine BMD (R=0.245, *p*=0.013), total hip BMD (R=0.197, *p*=0.048), T value of the total hip BMD (R=0.200, *p*=0.044), femoral neck BMD (R=0.205, *p*=0.018), and T value of the femoral neck BMD (R=0.243, *p*=0.046). However, it was negatively associated with β-CTX level (R=−0.309, *p*<0.001). Serum FGF19 level was positively correlated to femoral neck BMD (R=0.197, *p*=0.037), total hip BMD (R=0.235, *p*=0.013), and T value of the total hip BMD (R=0.193, *p*=0.041), but not to β-CTX level (R=−0.350, *p*<0.001).

## DISCUSSION

In this study, we evaluated the changes in serum bile acid and FGF19 levels in Chinese women with postmenopausal osteoporosis, and the relationship between BMD and bile acid metabolism was assessed. The bile acid and FGF19 levels were significantly lower in patients with postmenopausal osteoporosis than in healthy controls. A positive correlation was observed between serum bile acid level and BMD in postmenopausal women.

Only few relative studies have focused on the association between bile acid and bone metabolism. An animal study has found that bone loss in TGR5-/- knockout mice is more rapid than that in normal mice after aging or ovariectomy, and this even can be attributed to the regulation of osteoclast generation by TGR5 via the AMP-activated protein kinase signaling pathway. Another study has found that the BMD in FXR knockout mice decreases rapidly (up to 4.3%-6.6%) from 8 to 20 weeks; however, the administration of chenodeoxycholic acid or FXR receptor agonist can promote osteoblast differentiation and inhibit osteoclast differentiation, thereby resulting in increased BMD (11). According to a recent study, FXR agonists could promote the expression of osteogenesis-related genes, including those regulating bone sialoglycoprotein, osteocalcin, osteopontin, and alkaline phosphatase (12). To date, relevant studies are mostly based on basic research, and the serum bile acid levels of patients with osteoporosis have not been assessed in previous studies. This study found that the serum total bile acid level was significantly lower in women with postmenopausal osteoporosis and osteopenia than in healthy controls, which is consistent with the results of previous basic research. This finding also indicates that bile acid plays an important role in bone metabolism based on clinical evidence.

The specific mechanism underlying the effect of bile acid on bone metabolism is not clearly elucidated. FGF19 is the downstream molecule of bile acid. In the small intestine, bile acid is reabsorbed into the small intestine cells and binds to and activates FXR to induce the upregulation of FGF19 (13). FGF19 enters the blood circulation after it is secreted by the intestinal tract, and it plays an important role in metabolic regulation, such as controlling bile acid metabolism, gallbladder filling, and blood glucose; promoting energy metabolism; and reducing weight (5,14-16). However, the effect of FGF19 on bone metabolism is not fully elucidated. In addition, bile acid can activate TGR5 on the surface of small intestine cells and mediate the secretion of GLP-1 and GLP-2 by the endocrine cells in the intestine (17,18). Several studies have shown that GLP-1 can increase bone density and improve bone mass by increasing the number of osteoblasts and upregulating the expression of bone formation-related genes (19-22). Our study has found that the serum FGF19 levels were lower in patients with osteoporosis and osteopenia than in healthy controls, which is consistent with the results of the above mentioned studies. Thus, the decrease in bile acid synthesis may lead to a reduction in GLP-1 levels via the downregulation of FGF19 expression. However, further studies must be conducted to validate such notion. In addition, a decrease in bile acid synthesis can cause gastrointestinal abnormalities via the downregulation of FGF19 expression, which can then result in a reduction in BMD.

We found that serum total bile acid and FGF19 levels were positively associated with the BMD of the lumbar spine and hip. Zhenxi Li et al. have found that the administration of SH-479, which is a bile acid receptor agonist, to mice with postmenopausal osteoporosis increases BMD and improves bone microstructure (8). Moreover, the use of bile acid was found to be an effective treatment for type 2 diabetes, hyperlipidemia, obesity, and other diseases. Thus, bile acid may be a new therapeutic target for postmenopausal osteoporosis (23,24). However, a more in-depth research must be conducted to validate further the specific mechanism underlying the effect of bile acid on bone metabolism and its therapeutic effects.

This study had some limitations. First, the sample size was relatively small, and the number of participants in the control group was smaller than that in the osteoporosis and osteopenia groups. Second, since FXR and TGR5 are membrane receptors and are not expressed in the serum, changes in the expression of FXR and TGR5 in patients with osteoporosis could not be assessed. Third, this was a clinical study. Thus, the interference in bile acid detection in individuals with biliary tract diseases, abnormal glucose levels, lipid metabolism disorders, and other conditions could not be eliminated. Lastly, the study was conducted on Chinese patients only.

In summary, this cross-sectional study has found that the levels of serum bile acid and FGF19 were significantly lower in women with postmenopausal osteoporosis and osteopenia than in healthy women. Serum total bile acid and FGF19 levels were positively associated with BMD. Our findings indicate that the bile acid metabolic pathways can be new therapeutic targets for postmenopausal osteoporosis.

## AUTHOR CONTRIBUTIONS

Zhao YX, Zhuang XH, and Liu J designed the study. Zhao YX, Song YW, Zhang L, and Wang XM conducted the investigation and collected data. Wu F and Liu J performed the statistical analysis. Zhao YX and Song YW and Zheng FJ wrote the main manuscript. All authors reviewed and edited the manuscript and approved the final version.

## Figures and Tables

**Table 1:** Demographic characteristics of the participants.

	Osteoporosis group	Osteopenia group	Control group	*p*-value
number	52	75	23	
age (years)	59.8±3.8	58.6±4.4	58.4±4.5	0.220
height (cm)	157.9±5.4	159.1±4.1	158.4±3.7	0.354
weight (kg)	57.1±8.5	63.2±7.6	67.0±6.8	0.221
BMI (kg/m^2^)	22.9±2.8	25.0±2.9	26.7±2.7	0.109
TG (mmol/L)	1.32±0.68	1.50±1.07	1.28±0.59	0.397
TC (mmol/L)	4.86±0.73	5.03±0.86	5.19±0.95	0.246
HDL-C (mmol/L)	1.67±0.44	1.67±0.33	1.71±0.35	0.880
LDL-C (mmol/L)	2.56±0.78	2.58±0.77	2.71±0.79	0.707

BMI: body mass index; TG: triglyceride; TC: total cholesterol; HDL-C: high-density lipoprotein cholesterol; LDL-C: low-density lipoprotein cholesterol.

**Table 2:** Comparison of bone turnover biomarker levels and BMD between the three groups.

	Osteoporosis group	Osteopenia group	Control group	*p*-value
Number	52	75	23	
P1NP (pg/ml)	13.87±5.84	12.60±4.54	11.66±3.31	0.184
β-CTX (pg/ml)	572.3±154.4** Δ	543.6±96.3*	479.4±83.2	0.020
Grip strength (kg)	25.1±4.9	27.1±5.5	25.1±5.1	0.083
LS BMD (g/cm^2^)	0.717±0.102** ΔΔ	0.900±0.101**	1.033±0.066	**<0.001**
LS BMD T score	-3.048±0.812** ΔΔ	-1.280±0.877**	-0.087±0.578	**<0.001**
FN BMD (g/cm^2^)	0.603±0.076** ΔΔ	0.709±0.070**	0.807±0.048	**<0.001**
FN BMD T score	-2.354±0.505** ΔΔ	-1.310±0.426**	-0.302±0.127	**<0.001**
TH BMD (g/cm^2^)	0.722±0.083** ΔΔ	0.853±0.089**	0.958±0.096	**<0.001**
TH BMD T score	-2.281±0.665** ΔΔ	-1.252±0.653**	-0.378±0.416	**<0.001**

*,**, and ΔΔ indicate significant differences between the two groups after a pairwise comparison.

* *p*<0.05 compared with the control group;

** *p*<0.01 compared with the control group, and

ΔΔ *p*-value<0.01 compared with the osteopenia group.

P1NP: procollagen type I N-peptide; β-CTX: cross-linked C-terminal telopeptide of type I collagen; BMD: bone mineral density; LS: lumbar spine; FN: femoral neck; TH: total hip.
